# The Principles and Practice of Modern Surgery

**Published:** 1900-07

**Authors:** 


THE PRINCIPLES AND PRACTICE OF MODERN SURGERY. For the use of Students and Practitioners of Medicine and Surgery. By John B. Roberts, M. D., Professor of Anatomy and Surgery in the Philadelphia Polyclinic; Mütter Lecturer on Surgical Pathology of the College of Physicians of Philadelphia. New (2d) and revised edition. In one very handsome octavo volume of 838 pages, with 474 engravings and 8 plates in colors and monochrome. Cloth, $4.25, net; leather, $5.25, net. Philadelphia: Lea Brothers & Co.

This is a single volume work, and is sufficiently elaborate to meet the demands of students and general practitioners everywhere. The first edition was soon exhausted, which shows how well it has met the needs of medical men. This, the second edition, has been thoroughly revised and in many particulars it has been richly improved. Modern pathology and asepsis have received the closest attention. The author is one of the best known surgeons in America, and what he has written has always been received with enthusiastic interest the world over. T. J. B.

